# Thinking outside the box: Axillary to superior mesenteric artery bypass for chronic mesenteric ischemia

**DOI:** 10.1016/j.jvscit.2025.101929

**Published:** 2025-07-16

**Authors:** Austin R. Nelson, Justin M. Robbins, Jonathan Velasco

**Affiliations:** a Boonshoft School of Medicine, Department of Surgery, Wright State University, Dayton, OH; b Department of Vascular Surgery, Kettering Health Main Campus, Kettering, OH

**Keywords:** SMA bypass, Extra-anatomic bypass, Chronic mesenteric ischemia

## Abstract

Endovascular and open approaches are available for patients with chronic mesenteric ischemia. Axillary to superior mesenteric artery bypass has been described sparsely in the vascular literature. We describe a successful axillary to superior mesenteric artery bypass in an 84-year-old female patient with extensive aortoiliac disease precluding traditional approaches and performed in a community vascular surgery setting.

Chronic mesenteric ischemia is a visceral manifestation of peripheral vascular disease. Symptoms are often nonspecific and include nausea, emesis, abdominal pain, food fear, and weight loss. The prevalence of occlusive disease is estimated to be 10% in adults older than 65 years, with few patients becoming symptomatic and requiring intervention.^1^ Generally, patients do not become symptomatic until two of the mesenteric vessels are at least 70% stenosed.^2^ Risk factors for chronic mesenteric ischemia are similar to peripheral vascular disease and include hypertension, hyperlipidemia, and tobacco use.^3^

Revascularization options include open and endovascular approaches. Balloon angioplasty with or without a stent is now the preferred first-line treatment.^4^ If the patient is not an endovascular candidate, open reconstruction is possible. Traditional open approaches include supraceliac aorta to SMA bypass or iliac to SMA bypass.^4^ There is a subset of patients who are unable to be treated with any of these options. We describe the fifth reported case of axillary to SMA bypass in a patient with extensive aortoiliac disease.

## Case report

**Fig 1:**
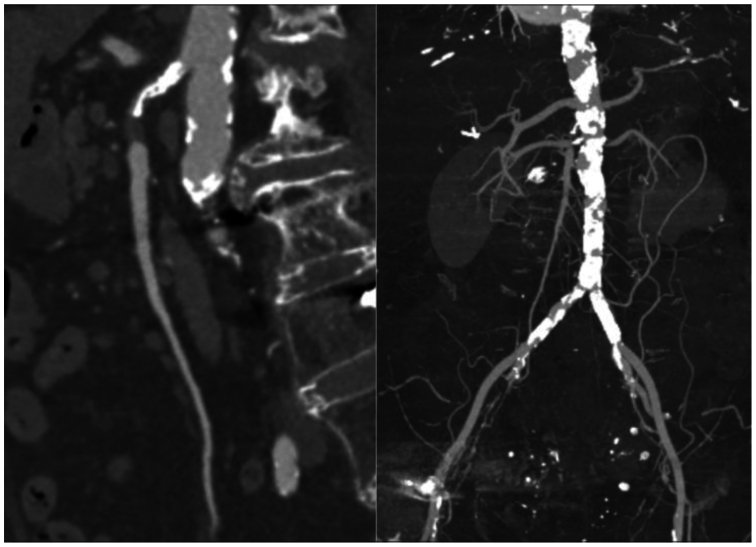
Preoperative imaging showing superior mesenteric artery (SMA) stenosis and aortoiliac disease.

The patient was an 84-year-old woman with a history of paroxysmal atrial fibrillation on warfarin, congestive heart failure, coronary artery disease, prior left iliac stent placement, open cholecystectomy, left inguinal hernia repair, laparoscopy for endometriosis, and chronic mesenteric ischemia. The patient was seen in the clinic with several months of weight loss, food fear, persistent nausea, and emesis. Workup included esophagogastroduodenoscopy and computed tomography angiography (CTA) showing aortoiliac calcifications with long segment occlusion of the SMA (Fig 1). Options for revascularization were discussed and a primary axillary to SMA bypass was offered.

The procedure began with a midline laparotomy. There were adhesions in the left upper quadrant. The base of the mesentery was identified and the SMA was palpated. The SMA was then dissected circumferentially until an appropriate segment was exposed. Left axillary artery exposure was performed via an infraclavicular incision, with proximal and distal control obtained. Anastomosis was performed in end-to-side fashion.

We tunneled an 8-mm polytetrafluoroethylene externally reinforced graft (W. L. Gore & Associates) from the left infraclavicular incision through the lateral subcutaneous tissue to the midaxillary line. An incision was made just distal to the left costal cartilage and a 10-mm trocar was tunneled under direct visualization superior to the colon. The trocar was removed and polytetrafluoroethylene was grasped and pulled into the abdominal cavity. A rent was made in the transverse colon mesentery so that the graft would lie in a retrocolic fashion. The graft was brought through the transverse colon mesentery and laid in approximation to the exposed SMA.

The anastomosis was created in a standard fashion with running 4-0 Prolene. Before completing the SMA anastomosis, the graft was flushed to de-air the graft.

**Fig 2:**
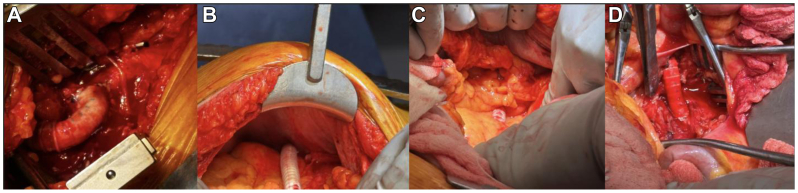
Intraoperative pictures showing **(A)** axillary anastomosis, **(B)** antecolic over left colon, **(C)** mesocolic tunnel, and **(D)** superior mesenteric artery (SMA) anastomosis.

The colon and viscera were replaced and the graft was evaluated. The graft entered the abdomen anterior to the omentum, which lay over the colon and posterior to the abdominal wall. The graft coursed through our retrocolic tunnel and into the mesentery of the small bowel to the anastomosis (Fig 2). We approximated the mesentery and peritoneum with Vicryl suture to cover the exposed graft.

**Fig 3:**
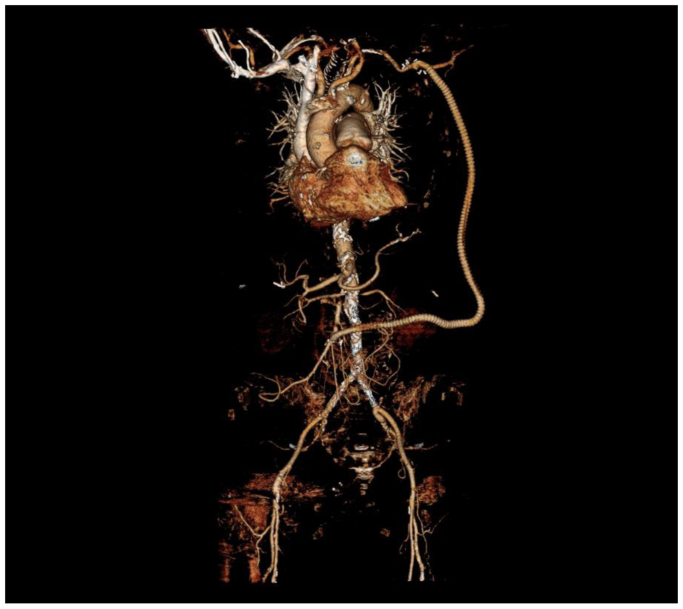
Postoperative computed tomography angiography (CTA) reconstruction.

Postoperatively, the patient was treated with our institution's vascular Enhanced Recovery After Surgery protocol, including clear liquids on the day of surgery. The day after, she was given a regular diet. She had complete resolution of abdominal pain and nausea with eating. A CTA was obtained postoperatively to provide baseline imaging (Fig 3). She was discharged home on postoperative day 3 and restarted on warfarin and aspirin. The patient in this case report agreed to have their case details and images published. A signed consent form was obtained per institutional protocol.

## Discussion

Traditional open approaches, such as antegrade aortomesenteric bypass or retrograde bypass using the infrarenal aorta or iliac arteries, have demonstrated excellent outcomes, with symptom improvement rates reported at 90% to 100%.^3^^,^^4^ Endovascular treatments have become the mainstay owing to high rates of technical success. These treatments are preferred in both low-risk and high-risk patients.^3^ However, there can be higher rates of recurrence, and these procedures are often impractical in patients with severe or diffuse disease.^5^

We explored several options for our patient, including direct aortic reconstruction, in either a supraceliac or infrarenal fashion; however, this procedure was not possible owing to severe aortic disease. We considered left iliac to SMA bypass, but the patient had prior stent placement, with evidence of severe in-stent stenosis and extensive surrounding disease. We felt that any attempt at bypass lower on the iliac could compromise blood flow to the lower extremity or the newly created bypass. Retrograde stenting was considered, but the vessel was completely occluded with ostial calcification and was dependent on collateral flow from the pancreaticoduodenal branch. Finally, it was felt that axillary to SMA bypass would be the best course of action.

The initial description of axillary to SMA bypass included a midline approach tunnelling deep to the pectoralis, along with placing a tongue of omentum in hopes of preventing bowel complications.^6^ Additional cases used a medial, subpectoral approach.^7^^,^^8^ A recent report discussed a similar approach using a left axillary anastomosis followed by tunneling from the left abdomen to the retroperitoneal SMA.^9^

Leaning on our general surgery background, we felt tunneling the graft from the lateral abdomen would be an ideal approach, allowing for either a retrocolic or an antecolic tunnel. Our preference was to tunnel the bypass posterior to the left colon to allow the bypass to be almost completely in the retroperitoneum. We originally planned to mobilize the white line of Toldt to keep the entire graft retroperitoneal. However, the patient had significant adhesion of the colon to the abdominal wall, likely from a prior intra-abdominal surgery, and we did not want to cause iatrogenic injury. We opted to tunnel the graft intraperitoneally and through the mesentery with a 1.5-cm defect at the base of the transverse colon mesentery. There is a theoretical risk of internal hernia; however, the peritoneum and mesentery over the graft were sutured closed, decreasing this risk.

Postoperatively, CTA was obtained to provide baseline imaging. The patient was seen 90 days after surgery with well-healing incisions and resolution of postprandial pain. A left chest wall seroma was noted. but was asymptomatic. We will monitor the graft with duplex ultrasound examinations at 6 months and 1 year postoperatively. The patient continues on her previously prescribed anticoagulation and antiplatelet therapy.

We describe the fifth reported case of axillary to SMA bypass and the third case with long-term follow-up. We demonstrate the additional effectiveness and safety of this extra-anatomic bypass, adding to the growing volume of literature to support the use of this bypass.

## Conclusions

This case demonstrates how using a multidisciplinary surgical approach to a complex patient can result in the best outcome for the patient. This further describes axillary to SMA bypass as a viable and underused option for patients with chronic mesenteric ischemia who cannot be treated with the usual approaches.

## Disclosures

None.
